# Use the right kidney contour as a landmark in adrenal vein sampling

**DOI:** 10.1371/journal.pone.0263945

**Published:** 2022-09-29

**Authors:** Jun Qian, Yun Du, Gang Yang, Yuanqing Yao, Bo Xiong, Shunkang Rong, Weiran Dai, Yonghong Jiang, Que Zhu, Changming Deng, Dichuan Liu, Jing Huang

**Affiliations:** Department of Cardiology, The Second Affiliated Hospital of Chongqing Medical University, Chongqing, China; Military Institute of Science and Technology, BANGLADESH

## Abstract

Adrenal Vein Sampling (AVS) is the gold standard for categorizing primary aldosteronism (PA). However, catheterization of the right adrenal vein (RAV) is challenging due the small size and variable location. This study aims to explore the relationship between the RAV orifice and the right kidney contour (RKC) on fluoroscopy, thus evaluating the potential of use the RKC as an anatomic marker for localizing RAV. Imaging data of 107 PA patients with successful bilateral AVS were retrospectively analyzed. Based on the body mass index (BMI), all patients were divided into the Normal Group (BMI < 24 kg/m^2^), Overweight Group (24 kg/m^2^ ≤ BMI < 28 kg/m^2^) and Obese Group (BMI ≥ 28 kg/m^2^). At the anterior view, the height level of RAV orifice was determined relative to vertebral bodies and disks. The distance from the RAV orifice to the upper edge of RKC was measured manually. The RAV orifice height level was mainly distributed from vertebral T11 to T12 (90.6%), and tended to be higher in patients with a larger BMI. The mean distance from the RAV orifice to the upper edge of RKC was 13.9±7.8mm, and had no difference among Normal group (n = 53, 14.1±8.2mm), Overweight group (n = 39, 13.7±8.0mm), and Obese group (n = 15, 13.9±5.5mm) (*p* = 0.981). Based on these findings, the RKC might be used as a landmark for localizing RAV on fluoroscopy, which is conductive to narrow down the exploration range and increase the success rate of RAV catheterization.

## Introduction

Primary aldosteronism (PA) is the most common detectable cause of secondary hypertension. Unilateral aldosterone-producing adenoma and bilateral idiopathic hyperaldosteronism are the main subtypes of PA, which are treated by different therapeutic approaches. CT or MRI examination may not be unreliable to distinguish unilateral from bilateral subtype. Thus, adrenal vein sampling (AVS) has become the gold standard for localizing lesions [1, 2].

However, AVS is a technically demanding interventional procedures even in experienced institutions. Although the catheterization of the left adrenal vein is usually uncomplicated, sampling of the right adrenal vein (RAV) is often more challenge due to the small size and variable locations of the orifice [3]. In most cases, the missing catheterization on the right side is the leading cause of the failure of bilateral AVS.

The RAV orifice often lies between the 11^th^ and 12^th^ vertebral body, that can be considered as an anatomic landmark based on the imageology studies [4, 5]. Identifying the kidney contour on fluoroscopy might also contributes to localize the approximate location of the RAV [6]. However, the detail relationship of RAV orifice and the kidney contour had not been explored. Thus, this study aims to probe the location of the RAV orifice with respect to the right kidney contour (RKC), and evaluate the value of use the RKC as an anatomic marker for the RAV catheterization.

## Materials and methods

### Patients

The present study was approved by the hospital institutional review board. The need for informed consent was waived by our hospital institutional review board. The digital subtraction angiography (DSA) images and other medical records of consecutive PA patients receiving AVS from Sep 2018 to May 2021 were retrieved from medical record database server of our hospital. All data were obtained from medical records in a fully anonymized and de-identified manner prior to analysis. None of the researchers had access to identifying information. Personal identification numbers were scrambled to protect patients privacy.

### AVS procedures

Prior to AVS, antihypertensive drugs and mineralocorticoid receptor antagonists (MRAs) were withdrawn for at least four weeks, if possible, or replaced with Calcium channel blockers (CCBs) and/or α receptor blocks based on individualized blood pressure.

The procedure was carried out between 8:00 am and 12:00 pm via antecubital vein approach. Two types of catheters were used for AVS in present study (5F MP, and 5F TIG). The 5F MP catheter was used for the RAV sampling, while the 5F TIG catheter was used for the left adrenal vein sampling as previously reported [7]. In detail, we first use the 5F MP catheter for probing the RAV. Probing was initiated on the right wall of the inferior vena cava (IVC) and the catheter advanced “up and down” to locate the orifice of the RAV. If the orifice is located along the probing line, the catheter will “drop in”. if not, the catheter was rotated backward gradually and the above steps were repeated until successful adrenal vein cannulation. The position of the catheter tip was confirmed by gently injecting a small amount of diluted contrast to reveal the angiography of the catheterized vein. Adrenal venograms at the anterior and lateral view were obtained at expiration period. AVS was considered as successful if the cortisol level in the sampled vein was two times higher than that in the IVC.

### Angiography imaging analysis

Based on the BMI, patients were divided into the Normal Group (BMI < 24 kg/m^2^), Overweight Group (24 kg/m^2^ ≤ BMI < 28 kg/m^2^) and Obese Group (BMI ≥ 28 kg/m^2^) according to the Consensus of Chinese Expert on Nutritional Medical Treatment for Overweight/Obesity in 2016 [8].

Two investigators (Dr Qian and Dr Du) independently reviewed and analyzed all imaging data using the Sante Dicom Viewer Free (V 5.3) software. The cranio-caudal level of the RAV orifice was determined relative to vertebral bodies and disks. The vertical distance of the RAV orifice to the upper edge of RKC was manually measured in all patients.

### Statistical analysis

Normally or approximately normally distributed measurement data were expressed as mean±standard deviation. Enumeration data were expressed as percentage (%). The distance between the RAV orifice and the upper edge of RKC among different groups was compared by one-way ANOVA. The difference of RAV height level between different groups was evaluated by chi-square test or Fisher’s exact test. Differences were considered as statistically significant if the *p* value was<0.05.

## Results

### Patients characteristics

The digital subtraction angiography (DSA) and other medical records of 116 consecutive PA patients were retrospectively reviewed. Based on the predetermined exclusion criteria, 9 patients were excluded due to the right AVS did not meet the selectivity criteria (Select Index≥2). One case was mistakenly catheterized into hepatic lobular vein. One case was failed due to the catheter dislocation during AVS. The remaining 7 cases were showed a common trunk of RAV and accessory hepatic vein (AHV), and the cortisol concentration might be diluted by the hepatic blood. Thus, a total of 107 patients with successful bilateral AVS were enrolled.

The characteristics of enrolled patients were shown in **Table 1**, involving 57 males and 50 females with a mean age of 43.7±11.2 years and mean plasma aldosterone-to-renin ratio (ARR) of 49.6±13.3{(ng/dL)/[μg/(L·h)]}. The AVS results in 107 successful cases revealed unilateral hyper-aldosteronism in 61 (57%) of patients. In the right adrenal, the mean aldosterone/cortisol level was 5150±1396ng/dL/1030±343ug/dL, and 955±291 ng/dL/1223±357 ug/dL with (n = 34) and without hyper-aldosteronism (n = 27), respectively.

**Table 1:** Patients characteristics.

Number of patients	107
Age	43.7±11.2
Male	57(53.3%)
BMI (Kg/m^2^)	24.6±2.7
ARR{(ng/dL)/[pg/(ml·h)]}	49.6±13.3
Serum Potassium (mmol/L)	3.7±0.9
24h MSBP (mmHg)	149±27
24h MDBP (mmHg)	89±13

BMI: Body Mass Index, ARR: aldosterone-to-renin ratio, MSBP: Mean Systolic Blood Pressure, MDBP: Mean Diastolic Blood Pressure.

### Imaging data analysis

A common trunk of the RAV with an accessory hepatic vein was found in 2/107 (1.9%) patients. The RAV orifice was located between T10 and L1 in all patients, in which, in 90.6% of the patients was located between the T11 to T12. Meanwhile, higher BMI value was tended correlate with a higher RAV orifice level (**Table 2**).

**Table 2:** The location of RAV orifice relative to vertebral bodies and disks (n %).

Group	n	Above T11+T11	Disk of T11-T12	T12+Below T12	χ^2^	*p*
Normal	53	12(22.6%)	9(17.0%)	32(60.4%)	11.572	0.018
Overweight	39	14(35.9%)	11(28.2%)	14(35.9%)		
Obese	15	9(60.0%)	3(20.0%)	3(20.0%)		

The difference between groups was tested by Fisher’s exact test.

**Fig 1:**
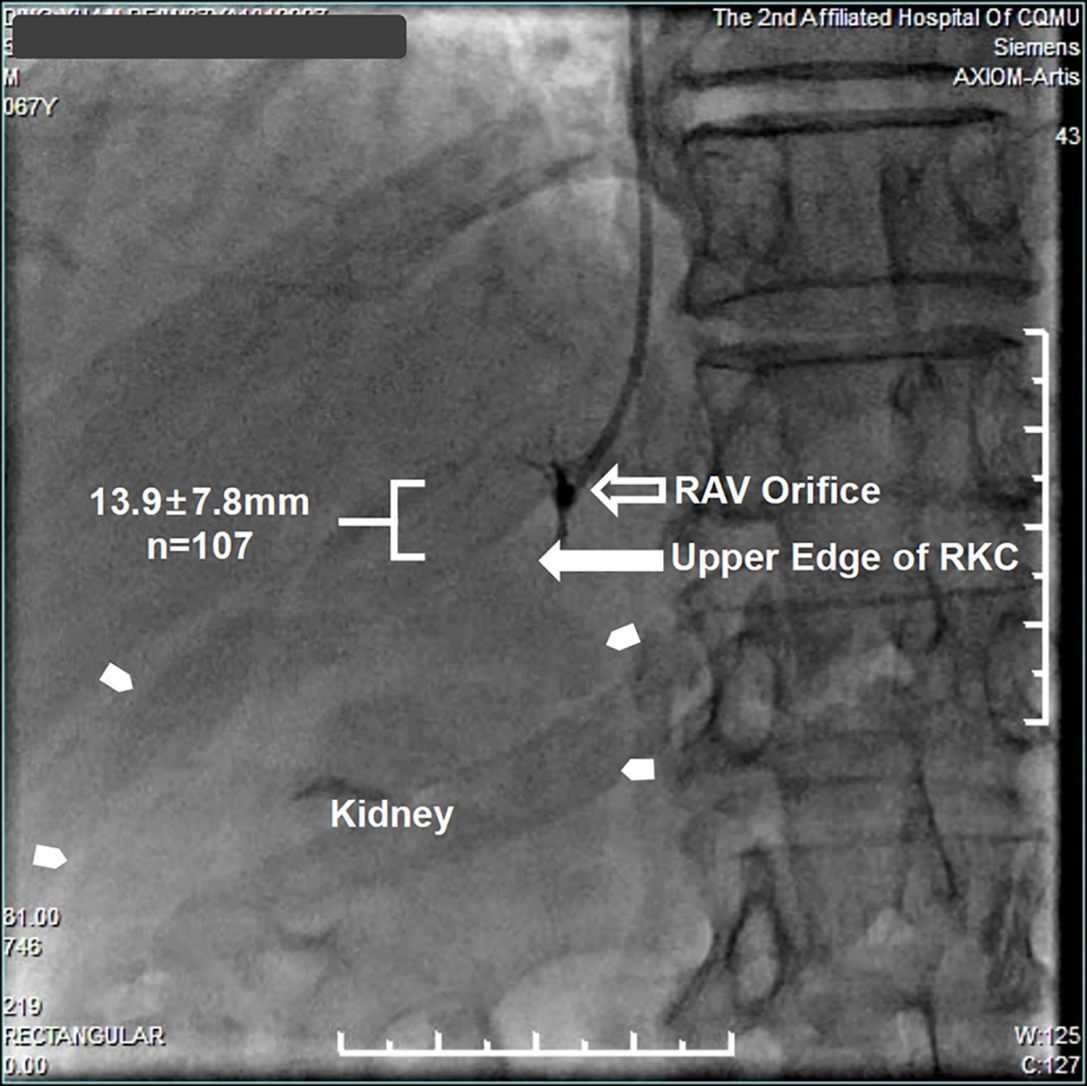
Relationship of the RAV orifice and RKC under fluoroscopy. The mean distance from the RAV orifice (**open arrow**) to the upper edge of RKC (**solid arrow**) was 13.9±7.8mm in 107 PA patients. The **tiny arrow head** indicated the RKC. **RAV: Right Adrenal Vein, RKC: Right Kidney Contour**.

**Fig 2:**
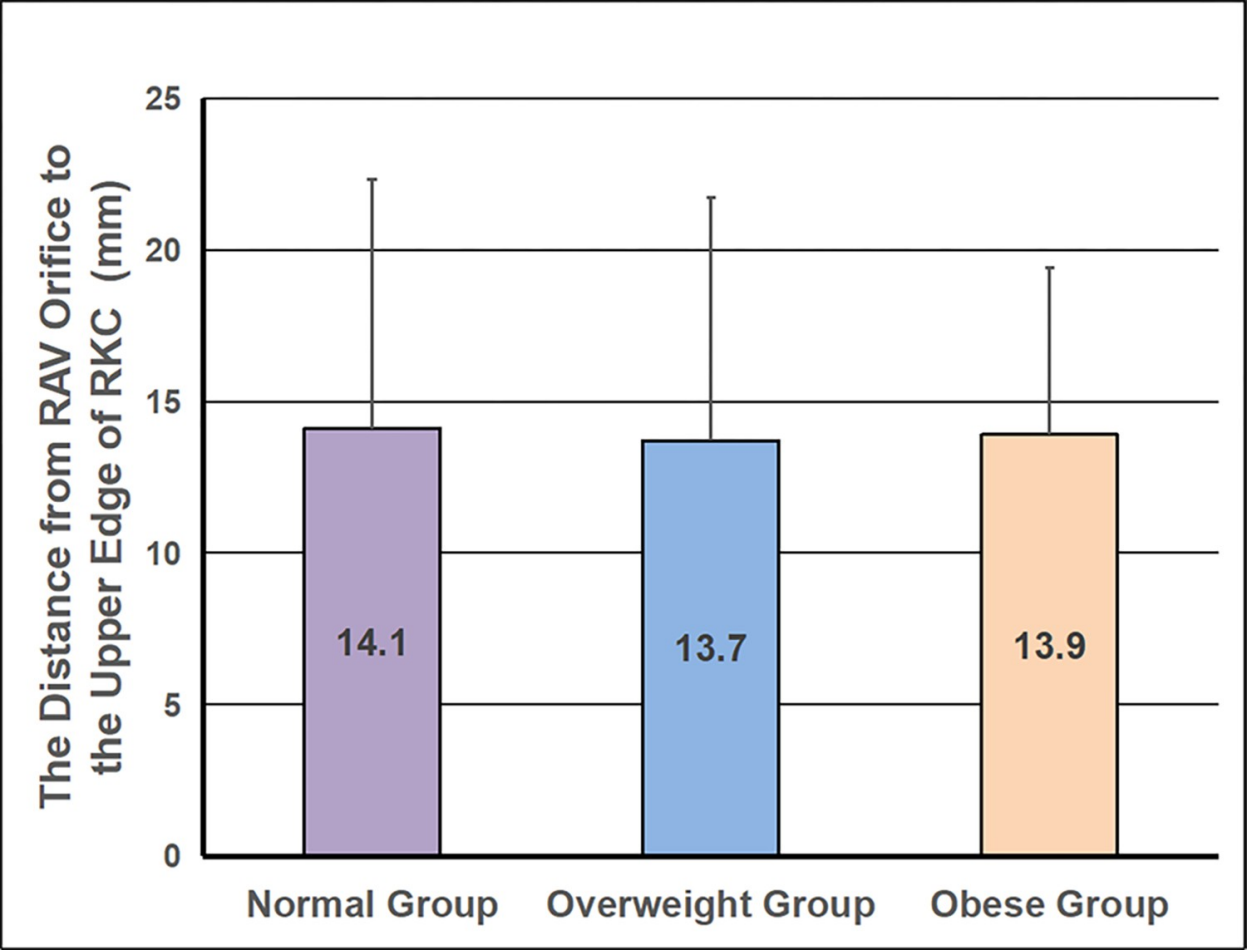
The distance from the RAV orifice to the upper edge of RKC in different groups. The mean distance from the RAV orifice to the upper edge of RKC had no difference among Normal group (n = 53, 14.1±8.2mm), Overweight group (n = 39, 13.7±8.0mm), and Obese group (n = 15, 13.9±5.5mm) (one-way ANOVA, *p* = 0.981). **RAV: Right Adrenal Vein, RKC: Right Kidney Contour**.

The RKC could be visualized in 75/107 (70.1%) patients under X-ray scanning, which could be shown in the remaining patients by injecting a small amount of contrast medium (about 10-15ml). The mean distance from the RAV orifice to the upper edge of RKC was 13.9±7.8mm in all patients (**Fig 1**), and mainly (81.3%) ranged within 20 mm. No significant difference in the mean distance from the RAV orifice to the upper edge of RKC was detected among Normal group (n = 53, 14.1±8.2mm), Overweight group (n = 39, 13.7±8.0mm), and Obese group (n = 15, 13.9±5.5mm) (*p* = 0.981) **(Fig 2)**.

In the 9 patients with failed AVS procedure, the mean distance from the sampled vein orifice to the RKC was 15.36 (11.15, 23.13) mm. There showed no significant difference while compared to the RAV with successful AVS **(Wilconxon Rank Sum Test, Z value = 1.316, *P* = 0.188**).

## Discussion

AVS is the crucial step in the clinical management of PA, which distinguishes unilateral from bilateral adrenal sources of autonomous aldosterone secretion [1, 2]. However, the difficulty of AVS is illustrated by low reported success rates of selective cannulation, not even reaching 50% in some centers or registries, that mainly due to great anatomic variations of the RAV in the junction with postcava on the right side [9].

Great efforts have been made on enhancing the success rate of RAV sampling. The vertebral body is usually used as a landmark for localizing the RAV orifice. In the present study, we consistently demonstrated that the RAV orifice was mainly distributed from T11 to T12 as previously reported [4, 5]. However, such an approximate range seemed broad while comparing to the tiny RAV orifice. Pre-operative enhanced CT scans might contribute to demonstrate the localization of RAV orifice relative to the vertebral body [4, 10]. However, the vessel is sometimes blurred on CT imaging, and not all the vessel can be detected. The right AVS success rate could be arisen to 100% using C-arm CT [11], but the C-arm CT is not available in all medical centers and might increase the medical cost and radiation exposure to patients.

Kidney contour is also an easily recognized anatomical landmark under X-ray, which might be helpful for localizing the RAV [6]. In our experience, the RKC was visualized in almost all patients during AVS procedure. The mean distance between the RAV orifice and the upper edge of RKC was 13.9±7.8 mm, and most of them were ranged within 20 mm, that was partly consistent with the anatomical relationship of the kidney and the expected location of the adrenal gland. Although the RAV orifice tended to be higher in patients with a larger BMI as in the previous studies, that might be introduced by the different level of diaphragm [12, 13]. No significant difference in the distance from the upper edge of RKC to the RAV orifice was found among different BMI groups. Based on these findings, probing the RAV orifice nearby the upper edge of RKC could be a simple approach to assist the RAV catheterization, and the RKC might become a promising landmark, that help for narrowing down the probing range along the inferior vena cava.

A total of 9 patients showed low cortisol level (<2 times than IVC) in the right side sampling vessels. By reviewing the DSA data, the mean distance from the sampled vessel to the RKC was 15.36(11.15, 23.13)mm in this 9 cases. Although the distance was tend to be longer while compare with the RAV with a normal anatomic configuration (11.59(9.13, 17.13)mm). However, there was no significant statistical difference (*P* = 0.188). The relative small sample size might have affected the results. Therefore, only the successful AVS cases were included in the analysis in the study, and we will further explore the RAV localization with variant in future.

There are some other limitations to the study. First of all, it was a retrospective, single-center study with a relatively small sample size, especially in the Obese group. Secondly, we only described the relative localization range of RAV orifice to the kidney contour. Other veins, like the accessory hepatic vein, hepatic lobular vein, might also located in this range, that could influence the RAV catheterization in real practice. Thirdly, a common trunk of the RAV with an accessory hepatic vein has been reported in approximately 8%-24% of individuals [14], while there were only 2 variants in this study, that might result from only the successful AVS cases were included. Therefore, the present study might only be targeted at RAV with a normal anatomic configuration. By reviewing the DSA data, a total of 9 cases showed a common trunk of RAV and AHV in 116 patients, including 7 cases suffered a failed AVS procedure. The proportion of RAV-AHV common trunk in this study (approximate 8%) was approximate to many previous studies [15, 16], indicated that the result might be suitable for most patients by using RKC as an anatomic reference for AVS. However, it was still a main limitation of the present study. With the increase of AVS cases in future, we will also explore the RAV localization of such variant.

## Conclusion

In conclusion, our study depicted the relationship of RAV orifice and the RKC under fluoroscopy. Given the above findings, localization of RAV based on the RKC as an anatomical landmark could help to approximate predict the location of RAV in populations with different BMI. This might provide a more speedy and precise procedure with higher success rate, especially for less experienced hands.

## Supporting information

S1 File The distance from the RAV orifice to the upper edge of RKC (mm). (XLSX)

S2 File The location of RAV orifice relative to vertebral bodies and disks. (XLSX)
